# Scavenger Receptor A1 Mediates the Uptake of Carboxylated and Pristine Multi-Walled Carbon Nanotubes Coated with Bovine Serum Albumin

**DOI:** 10.3390/nano11020539

**Published:** 2021-02-20

**Authors:** Mai T. Huynh, Carole Mikoryak, Paul Pantano, Rockford Draper

**Affiliations:** 1 Department of Chemistry and Biochemistry, The University of Texas at Dallas, 800 West Campbell Road, Richardson, TX 75080-3021, USA; Mai.t.Huynh@utdallas.edu (M.T.H.); pantano@utdallas.edu (P.P.); 2 Department of Biological Sciences, The University of Texas at Dallas, 800 West Campbell Road, Richardson, TX 75080-3021, USA; mikoryak@utdallas.edu

**Keywords:** carbon nanotube, macrophages, scavenger receptor, phagocytosis, protein corona, bovine serum albumin

## Abstract

Previously, we noted that carboxylated multi-walled carbon nanotubes (cMWNTs) coated with Pluronic^®^ F-108 (PF108) bound to and were accumulated by macrophages, but that pristine multi-walled carbon nanotubes (pMWNTs) coated with PF108 were not (Wang et al., *Nanotoxicology* **2018**, *12*, 677). Subsequent studies with Chinese hamster ovary (CHO) cells that overexpressed scavenger receptor A1 (SR-A1) and with macrophages derived from mice knocked out for SR-A1 provided evidence that SR-A1 was a receptor of PF108-cMWNTs (Wang et al., *Nanomaterials* (Basel) **2020**, *10*, 2417). Herein, we replaced the PF108 coat with bovine serum albumin (BSA) to investigate how a BSA corona affected the interaction of multi-walled carbon nanotubes (MWNTs) with cells. Both BSA-coated cMWNTs and pMWNTs bound to and were accumulated by RAW 264.7 macrophages, although the cells bound two times more BSA-coated cMWNT than pMWNTs. RAW 264.7 cells that were deleted for SR-A1 using CRISPR-Cas9 technology had markedly reduced binding and accumulation of both BSA-coated cMWNTs and pMWNTs, suggesting that SR-A1 was responsible for the uptake of both MWNT types. Moreover, CHO cells that ectopically expressed SR-A1 accumulated both MWNT types, whereas wild-type CHO cells did not. One model to explain these results is that SR-A1 can interact with two structural features of BSA-coated cMWNTs, one inherent to the oxidized nanotubes (such as COOH and other oxidized groups) and the other provided by the BSA corona; whereas SR-A1 only interacts with the BSA corona of BSA-pMWNTs.

## 1. Introduction

The interaction of engineered nanoparticles (ENPs) with cells is influenced by a corona of macromolecules that deposit on the ENP surface from the surrounding biological fluid. What macromolecules (often proteins) adhere to the ENP depends on the properties of the macromolecules and on the ENP surface structure, charge, hydrophobicity, and geometry [1,2,3,4]. Corona components may provide dominant features controlling the interaction of ENPs with specific cell surface binding sites, often followed by ENP internalization and a subsequent response by the cells. Understanding what corona components are present on an ENP and how they interface with cells is thus important to provide rational approaches for promoting positive responses, such as targeted drug delivery, or mitigating negative responses, such as toxicity. However, understanding ENP coronas is challenging because the potential corona components in complex biological environments are diverse and the properties of ENP surfaces vary widely. Single-walled carbon nanotubes (SWNTs) and multi-walled carbon nanotubes (MWNTs) are ENPs whose production is increasing due to a wide variety of commercial applications [5,6,7,8]. Nevertheless, there is ample evidence that carbon nanotubes can be toxic to organisms and the environment, but how their coronas contribute to toxicity is not well understood [9,10,11]. 

We previously noted that carboxylated MWNTs (cMWNTs) coated with Pluronic^®^ F-108 (PF108) preferentially bind to and are accumulated by cells, whereas PF108-coated pristine MWNTs (pMWNTs) do not bind and are poorly accumulated [12]. This suggested that surface receptors on macrophages selectively bind cMWNTs but not pMWNTs. Class A scavenger receptors (SR-As) are membrane glycoproteins that bind polyanionic compounds and modified proteins [13,14,15], and several observations in the literature implicate SR-As as potential carbon nanotube receptors. For example, there is evidence that SWNTs coated with bovine serum albumin (BSA) are targeted to SR-As [16]. There are also numerous reports where antagonists of class A-type 1 scavenger receptors (SR-A1s) affect cell responses to MWNTs: The accumulation of cMWNTs by RAW 264.7 macrophages correlated with the extent of carboxylation and was inhibited by the SR-A1 antagonist dextran sulfate [17]; the rate of apoptosis induced by MWNTs could be reduced by treating the cells with poly I, another SR-A antagonist [18]; and the accumulation of FITC-BSA-coated MWNTs by THP-1 macrophages was inhibited by the SR-A antagonist fucoidan [19]. In addition, Hirano et al. found that MWNTs suspended in the surfactant Pluronic^®^ F-68 bind to MARCO (SR-A6) receptors on Chinese hamster ovary (CHO) cells overexpressing MARCO [20]. We also observed that dextran sulfate reduced the binding of PF108-coated cMWNTs by macrophages [12].

Recently, we reported that alveolar macrophages derived from SR-A1 knockout mice did not bind or accumulate PF108-cMWNTs whereas they were accumulated by CHO cells that ectopically expressed SR-A1 [21]—strong evidence that SR-A1 is a receptor for PF108-coated cMWNTs. An interesting feature of PF108-coated cMWNTs is that they bind strongly to cells in the absence of serum or any exogenous protein, suggesting that a protein corona is not required for cMWNT binding to SR-A1 [12]. Thus, some inherent structural feature of oxidized MWNTs, perhaps carboxyl groups, carbonyl groups, or hydroxyl groups, appear sufficient for interaction with SR-A1.

Herein, we replaced the PF108 coat with BSA and studied the interaction of cMWNTs and pMWNTs bearing a BSA corona with CHO cells that ectopically express SR-A1 and with RAW 264.7 cells that were deleted for SR-A1 using CRISPR-Cas9 technology. CHO cells expressing SR-A1, but not wild-type (WT) CHO cells, accumulated both BSA-coated cMWNTs and pMWNTs, but the amount of cMWNTs accumulated was 2–3 times more than pMWNTs. WT RAW 264.7 cells also accumulated approximately 2 times more BSA-coated cMWNTs than pMWNTs. Moreover, in binding studies with RAW 264.7 cells at 4 °C in the absence of serum, more BSA-cMWNTs than BSA-pMWNTs were bound. These data suggest that there are more binding sites on the RAW 264.7 cell surface for BSA-cMWNTs than BSA-pMWNTs. To assess what effect the absence of SR-A1 would have, the binding and accumulation of BSA-coated cMWNTs and pMWNTs to SR-A1 knockout RAW 264.7 cells at 4 °C in medium without serum and at 37 °C was measured. The amount of bound or accumulated BSA-MWNTs in the knockout SR-A1 cells was significantly decreased for both BSA-pMWNTs and BSA-cMWNTs compared to the WT RAW 264.7 cells. These observations suggest that pMWNTs coated with a BSA protein corona gain the capacity to bind SR-A1. Overall, BSA-cMWNTs have enhanced binding to SR-A1 above that observed with BSA-pMWNTs, emphasizing the differences between how BSA-coated cMWNTs and pMWNTs interact with receptors. Models to account for the differences are presented.

## 2. Materials and Methods 

### 2.1. Nanomaterials

The pMWNT (product 1236-YJS, lot 2015-041709) and cMWNT (product 1256-YJF, lot 2015-070510) powders were purchased from Nanostructured & Amorphous Materials, Inc. (Houston, TX). pMWNTs and cMWNTs were synthesized using a Fe/Co/Ni-catalyzed chemical vapor deposition process. Caution should be taken, and a fine particulate respirator and other appropriate personal protective equipment should be worn when handling dry MWNT powders. Both MWNT products were reported by the manufacturer to be >95% in purity and to contain MWNTs with outer diameters of 10–20 nm, inner diameters of 5–10 nm, and lengths of 0.5–2 µm. The cMWNT powder was oxidized using sulfuric acid and potassium permanganate and comprised 1.9–2.1% by weight carboxylic acid groups. Elemental analyses of MWNTs were performed using a previously described combustion analysis technique [22]. The combined carbon, hydrogen, nitrogen, sulfur, and oxygen elemental analyses of the pMWNTs and cMWNTs were 99.52% and 98.18%, respectively, indicative of MWNT powders that are essentially metal-free. An extensive physical and chemical characterization of the pMWNTs and cMWNTs powders appears elsewhere [23]. The major similarities of the pMWNTs and cMWNTs were their outer diameters (18 ± 3 nm and 19 ± 5 nm, respectively) and inner diameters (5.6 ± 1.3 and 5.7 ± 1.7 nm, respectively), as determined using transmission electron microscopy. The key difference was the presence of a carbonyl vibrational stretching mode associated with carboxyl groups in the infrared spectra of cMWNTs that was not observed in the pMWNT spectra.

### 2.2. Chemicals and Solutions

Dulbecco’s modified Eagle medium (DMEM) and Ham’s F-12K complete medium were purchased from Gibco (Grand Island, NY, USA), fetal bovine serum (FBS) from Atlanta Biologicals (Flowery Branch, GA, USA), Geneticin^®^ selective antibiotic G418 sulfate from Calbiochem (San Diego, CA, USA), and Accumax^TM^ from Innovative Cell Technologies (San Diego, CA, USA). SR-AI/MSR Alexa Fluor^®^ 488-conjugated antibody and rat IgG2B Alexa Fluor^®^ 488-conjugated Isotype Control were purchased from R&D Systems (Minneapolis, MN, USA). Bovine serum albumin (BSA), dextran sulfate (product # D6001), chondroitin sulfate (product # C9819), penicillin (10,000 U/mL), streptomycin (10 mg/mL), and all other chemicals were purchased from Millipore Sigma (Burlington, MA, USA). All chemicals were used as received. Deionized water (18.3 MΩ·cm) was obtained using a Milli-Q^®^ Integral water purification system (Billerica, MA, USA). Phosphate buffered saline (PBS; 0.8 mM phosphate, 150 mM NaCl, pH 7.4) was sterilized by autoclaving at 121 °C for 45 min. Stock solutions of 100 mg/mL BSA were prepared by dissolving 10 g of BSA in 100 mL of deionized water and adjusting the pH to 7.4. Working solutions of 0.10 mg/mL BSA were prepared by diluting stock BSA solutions with aqueous 10 mM HEPES (pH 7.4) and filtering the solutions through a 0.22-μm pore membrane; stock and working solutions of BSA were stored at 4 °C in the dark. 

### 2.3. Cell lines and Cell Culture

Abelson murine leukemia virus transformed RAW 264.7 macrophages were purchased from the American Type Culture Collection (ATCC^®^ TIB-71™; Manassas, VA, USA). A scavenger receptor A1 (SR-A1) knockout RAW 264.7 cell pool was purchased from Synthego Corporation (Silicon Valley, CA, USA). The cell pool was generated using CRISPR-Cas9 technologies with the guide RNA sequence CAGCAUCCUCUCGUUCAUGA. Synthego validated, via genome sequencing, that 70% of the SR-A1 knockout pool of RAW 264.7 cells had insertion(s) or deletion(s) between base pairs 41 and 42 of the SR-A1 gene. Because the site of alteration is at the beginning of the gene, expression of SR-A1.1, which is a splice variant of SR-A1, would also be affected. A dilution scheme was used to clone cells that did not express SR-A1 receptors on their surface. Serial dilutions of the SR-A1 knockout RAW 264.7 cell pool were plated in 96-well plates and incubated for 7 days. Cells that had arisen from a single colony were grown for several passages before selecting clones that lacked surface SR-A1 expression using immunofluorescence microscopy and flow cytometry. All RAW 264.7 cells and SR-A1 knockout RAW 264.7 cells were grown in DMEM supplemented with 1.5 mg/mL sodium bicarbonate, 10 mM HEPES (pH 7.4), and 10% (*v/v*) FBS.

Chinese hamster ovary (CHO) cells stably transfected with mouse SR-A1 cDNA (CHO[mSR-AI] cells) were generously provided by Professor Monty Krieger (Massachusetts Institute of Technology) [24]. The control WT CHO cell line for CHO[mSR-AI] cells were CHO-K1 cells (ATCC^®^ CCL-61™). All CHO cells were grown in Ham’s F-12K medium supplemented with 2.0 mg/mL sodium bicarbonate, 10 mM HEPES (pH 7.4), 10% (*v/v*) FBS, 100 units/mL penicillin, and 100 μg/mL streptomycin; the mSR-AI cells were additionally maintained under 0.25 mg/mL G418. The standard incubation conditions for all cell lines were 37 °C in a 5% CO_2_ and 95% air environment.

### 2.4. Preparation of BSA-MWNT Suspensions

**Scheme 1:**
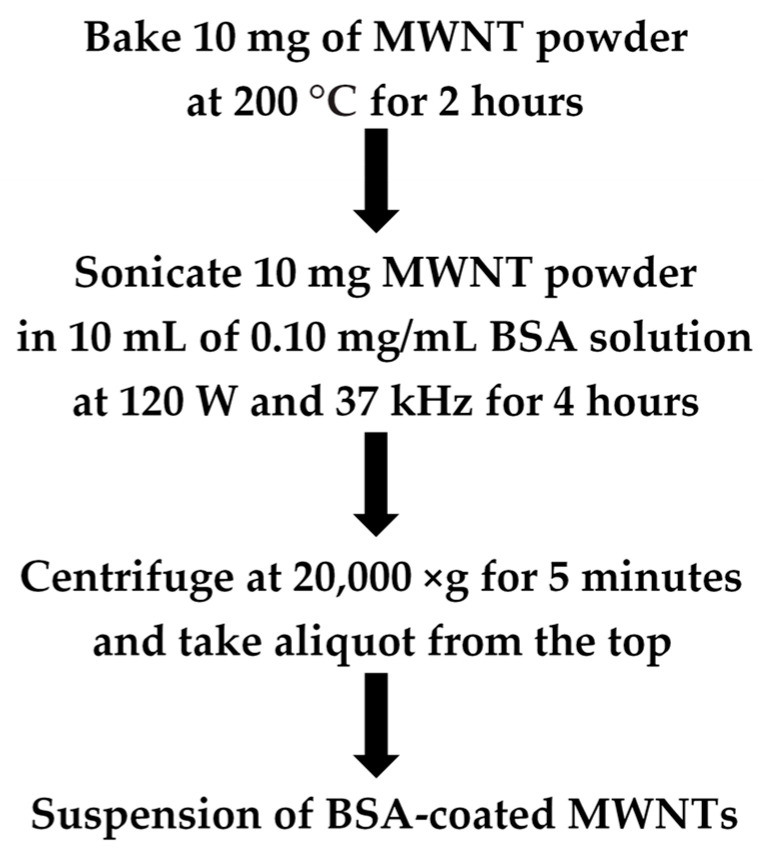
Preparation of purified BSA-coated MWNT suspensions by sonication and centrifugation.

The sonication and centrifugation protocol described in our previous works [12,25] was used with slight modifications to prepare purified BSA-coated MWNT suspensions, as summarized in Scheme 1. MWNTs were coated with BSA to match the albumin in the FBS used in growth media. A total of 10.0 mg of pMWNT or cMWNT powder was weighed into a pre-cleaned 20-mL glass vial and baked at 200 °C for 2 h to inactivate potential endotoxin contaminants [26]. Next, 10 mL of a 0.10 mg/mL BSA working solution was added to the vial and the mixture was sonicated. Specifically, a single vial was secured in a hanging rack and sonicated for 240 min using an ultrasonic bath sonicator (Elmasonic P30H; Elma Ultrasonic, Singen, Germany) that was operated at 120 W and 37 kHz in a 4 °C cold room. During sonication, the temperature of the bath water was maintained below 18 °C by using a refrigerated water bath circulator (Isotemp 1006S). After sonication, the solution was divided by transferring 1-mL aliquots into ten 1.5-mL centrifuge tubes. One of the 1-mL aliquots of each non-centrifuged BSA-pMWNT or BSA-cMWNT suspension was set aside as the standard suspension, and each standard solution was serially diluted with a 0.10 mg/mL-BSA working solution. The absorbance at 500 nm of the dilutions determined using a BioTek SynergyMx plate reader (Winooski, VT, USA) was used to construct pMWNT or cMWNT calibration curves. The remaining nine aliquots were centrifuged at 20,000 RCF for 5 min at 4 °C using an Eppendorf 5417R centrifuge to remove MWNT bundles and other impurities, as demonstrated in our previous work [27]. The top 900 µL from each supernatant was collected without disturbing the pellet and combined in a sterile vial to afford ~9 mL of a purified BSA-pMWNT or BSA-cMWNT suspension. The concentration of MWNTs in each purified suspension was determined using the measured absorbance at 500 nm and the calibration curves described above. Purified BSA-MWNT suspensions were stored at 4 °C in the dark.

### 2.5. Characterization of MWNT Suspensions

The particle size distributions, in terms of hydrodynamic diameter, of BSA-MWNT suspensions were determined by dynamic light scattering (DLS). In brief, aliquots of purified pMWNT or cMWNT suspensions were diluted 1:10 in a 0.10 mg/mL BSA working solution and analyzed using a 633-nm laser and a backscatter measurement angle of 173° (Zetasizer Nano-ZS 3600, Malvern Instruments, Worcestershire, UK). The instrument was calibrated with Polybead^®^ standards (Polysciences, Warrington, PA, USA) and ten consecutive 30-s runs were taken per measurement at 25 °C. The hydrodynamic diameter was calculated using a viscosity and refractive index of 0.8872 cP and 1.330, respectively, for deionized water, and an absorption and refractive index of 0.010 and 1.891, respectively, for MWNTs. Zeta potential values were also determined for purified BSA-coated MWNT suspensions that were diluted 1:10 with deionized water, medium with serum, or serum-free medium. In addition, DLS and zeta potential analyses were performed periodically on purified MWNT suspensions stored at 4 °C to detect any changes. Typically, MWNT suspensions were stable in storage for months, indicated by the lack of aggregates detected by DLS and constant zeta potential results.

### 2.6. Crystal Violet Cell Proliferation Assay

For the assays with RAW 264.7 cells, purified BSA-MWNT suspensions were first diluted with a freshly prepared 0.10 mg/mL-BSA working solution to a concentration twice the desired MWNT concentration to be tested. The diluted MWNT suspensions were then mixed 1:1 in equal volumes with 2X-concentrated medium that contained 3.0 mg/mL sodium bicarbonate, 20 mM HEPES (pH 7.4), 20% (*v/v*) FBS, 200 units/mL penicillin, and 0.2 mg/mL streptomycin. The result is a test medium with the same concentration of 10 mM HEPES and 10% FBS as the control medium. A total of ~3.5 × 10^4^ RAW 264.7 cells/well were seeded in 48-well plates and incubated at 37 °C overnight before the medium was replaced with freshly prepared control medium or test medium containing MWNTs and incubated for 24 h. At the end of the incubation, cells were washed 3 times with fresh medium, 2 times with PBS, air-dried, and fixed with 4% (*w/v*) paraformaldehyde in PBS. Cell proliferation was determined using a standardized crystal violet assay, as described in our previous work where it was demonstrated that MWNTs do not interfere with the assay [28].

### 2.7. Quantitation of MWNTs Extracted from Cell Lysates by SDS-PAGE

The SDS-PAGE method with optical detection [29], previously validated by a large-area Raman scan technique [12], was used for quantifying MWNTs extracted from RAW 264.7 cells or CHO cells. In brief, aliquots of known amounts of pMWNT or cMWNT standard suspensions, lysates of control cells, and lysates of cells treated with MWNTs were mixed with 5% 2-mercaptoethanol, 10% glycerol, 62.5 mM Tris-HCl, pH 6.2, and 2X-concentrated SDS sample loading buffer to a final concentration of 2% SDS, and boiled for 3 min. Samples at various dilutions and volumes were subsequently loaded into the wells of an SDS-polyacrylamide gel composed of a 4% stacking gel on top of a 10% resolving gel. An electric current was applied at a constant 100 V for 2 h. MWNTs in standard suspensions and in the lysates bind SDS in the sample loading buffer to become negatively charged and migrate toward the anode upon electrophoresis. The large aspect ratio of MWNTs prevents them from sieving through the pores of a 4% polyacrylamide gel mesh; thus, the MWNTs accumulate at the bottom of the sample loading well during electrophoresis and form a sharp dark band. Following electrophoresis, optical images of the gels were obtained using a flatbed scanner (HP Scanjet G3110, Hewlett Packard Enterprise, Fort Collin, CO, USA), and the pixel intensity of each dark band was quantified using ImageJ software (NIH ImageJ system, Bethesda, MD, USA). The known amount of MWNTs in the standards and their corresponding pixel intensities form a linear calibration curve that was used to determine the unknown amount of MWNTs in cell lysates, based on the pixel intensities of lysate bands loaded in the same gel as the standards. 

### 2.8. Accumulation of MWNTs by Cells at 37 °C

The following procedure was used to detect the accumulation of pMWNTs and cMWNTs by RAW 264.7 or CHO cells at 37 °C for 24 h. MWNT suspensions were first diluted in a freshly prepared 0.10 mg/mL BSA working solution to twice the desired final MWNT concentrations specified in the experiment. The diluted MWNT suspension samples were then mixed 1:1 with the appropriate 2X-concentrated medium. A total of ~3.5 × 10^5^ cells/well were seeded in 6-well plates and incubated in medium at 37 °C overnight to allow the cells to adhere to the plates. The medium was removed the next day and 2 mL of the appropriate freshly prepared control medium that contained no MWNTs or test medium that contained an MWNT suspension at a specified concentration was added to each well. Cells were incubated in a control or test medium at 37 °C for 24 h, as described in each experiment. At the end of the incubation, the control and test media were removed by aspiration and the cells were washed 3 times with fresh medium followed by 2 washes with PBS. Cells were then lifted off the well using 0.5 mL Accumax^TM^, transferred to a centrifuge tube, and the well was rinsed with 1.5 mL PBS that was subsequently added to the tube to make a final cell suspension of 2 mL/well/tube. Three aliquots of cell suspension, 100 µL each, were used to determine cell counts in each sample using a Beckman Coulter particle counter (Miami, FL, USA) and the cells in the remaining 1.7-mL cell suspension were collected by centrifugation at 1000× *g* for 5 min at 4 °C. The cells in the pellet were lysed in 200 µL of cell lysis buffer that contained 0.25 M Tris-HCl (pH 6.8), 8% (*w/v*) SDS, and 20% (*v/v*) 2-mercaptoethanol. To ensure complete lysis of the cells, the lysate samples were heated in a boiling water bath for 2 h and then stored at 4 °C. The amounts of MWNTs in the cell lysate samples were determined using the SDS-PAGE method, as described previously herein.

### 2.9. Surface Binding of MWNTs to Cells at 4 °C

To detect and compare the association of pMWNTs and cMWNTs to the surface of RAW 264.7 cells in the absence of endocytic or phagocytic activity, ~5.0 × 10^5^ RAW 264.7 cells/well were first seeded in 6-well plates and incubated in the appropriate medium at 37 °C overnight. Then, the cells were incubated in the appropriate serum-free medium for 2 h at 37 °C to deplete the serum in the cells. In order to incubate cells at a low temperature outside of the 37 °C incubator, the medium was replaced with the respective serum-free medium that additionally did not contain sodium bicarbonate. The 6-well plates were then placed on a shallow ice-water bath and incubated in a 4 °C cold room for 30 min. The appropriate 2X-concentrated, serum- and sodium bicarbonate-free medium was pre-chilled to 4 °C before mixing 1:1 with a MWNT suspension, such that the final test medium contained MWNTs at the desired concentration specified in the experiment. After chilling down to 4 °C, the cells were incubated for 1 h at 4 °C with the appropriate pre-chilled serum- and sodium bicarbonate-free medium that did not contain MWNTs (control), or test serum- and sodium bicarbonate-free medium that contained a MWNT suspension at the specified final MWNT concentration. Because phagocytosis and endocytosis are blocked at low temperature, MWNTs in the test medium were free to interact with cell surface components without subsequently entering the vacuolar compartment of the cells. After incubation, the cells were washed, harvested, and the subsequent procedures for cell counting and lysate preparation were followed, as described in the previous sections. The amounts of cell-surface bound MWNTs in the cell lysate samples were determined using the SDS-PAGE method, as described previously herein.

### 2.10. Dissociation of Bound BSA-cMWNTs and BSA-pMWNTs from RAW 264.7 Cells at 4 °C

MWNTs suspended in a 0.10 mg/mL BSA working solution were mixed with an equal volume of 2X-concentrated, serum- and sodium bicarbonate-free medium to give a final MWNT concentration of 100 µg/mL. Equivalent number of RAW 264.7 cells were seeded in 6-well plates and incubated at 37 °C under standard cell culture conditions for 24 h prior to the experiment. Next, the cells were pre-incubated with serum-free medium (in the absence of MWNTs) for 2 h at 37 °C to deplete the serum in the cells. The cells were then pre-chilled to 4 °C and incubated at 4 °C for 1 h in serum- and sodium bicarbonate-free medium that contained either BSA-pMWNTs or BSA-cMWNTs. Finally, the cells were then incubated with serum- and sodium bicarbonate-free medium for an additional 20, 40, 60, 90, or 120 min, and then washed 3 times with serum- and sodium bicarbonate-free medium, then 2 times with PBS. After incubation, surface-bound MWNTs were extracted and quantified by the SDS-PAGE method, as described previously herein.

### 2.11. Additive Binding Test for BSA-cMWNTs and BSA-pMWNTs to RAW 264.7 Cells

To determine whether BSA-cMWNTs and BSA-pMWNTs use independent surface binding sites, ~5.0 × 10^5^ RAW 264.7 cells/well were first seeded in 6-well plates and incubated in medium at 37 °C overnight. Cells were then incubated in a serum-free medium for 2 h at 37 °C to deplete the serum in the cells. Next, this medium was replaced with a serum-free medium that did not contain sodium bicarbonate. The 6-well plates were placed on a shallow ice-water bath and incubated in a 4 °C cold room for 30 min. A 2X-concentrated, serum- and sodium bicarbonate-free medium was pre-chilled to 4 °C before mixing 1:1 with a MWNT suspension such that the final test serum- and sodium bicarbonate-free medium contained 100 μg/mL MWNTs. After chilling to 4 °C, the cells were incubated with either BSA-cMWNTs or BSA-pMWNTs separately at 4 °C for 90 min or simultaneously with both ligands at 4 °C for 90 min. In a slightly different experimental design, the ligands were added sequentially, first BSA-cMWNTs for 45 min at 4 °C followed by washing the cells and the addition of BSA-pMWNTs, for 45 min at 4 °C for a total incubation time of 90 minutes. The order of the ligand addition was then reversed with another set of cells. The amounts of cell-surface bound MWNTs in the cell lysate samples were determined using the SDS-PAGE method, as described previously herein.

### 2.12. Surface Binding of MWNTs to RAW 264.7 Cells in the Presence of Dextran Sulfate, an SR-A1 Antagonist

To determine the effects of dextran sulfate on the association of pMWNTs and cMWNTs to the surfaces of RAW 264.7 cells, ~5.0 × 10^5^ RAW 264.7 cells/well were seeded in 6-well plates and incubated in medium at 37 °C overnight. Then, RAW 264.7 cells were incubated in serum-free medium for 2 h at 37 °C to deplete the serum in the cells. To incubate cells at low temperature outside of the 37 °C incubator, the serum-free medium was replaced with serum-free medium that did not contain sodium bicarbonate. The 6-well plates were then placed on a shallow ice-water bath and incubated in a 4 °C cold room for 30 min. A 2X-concentrated, serum- and sodium bicarbonate-free medium was pre-chilled to 4 °C before mixing 1:1 with a MWNT suspension followed by the addition of dextran sulfate (or chondroitin sulfate, a control that is not an SR-A1 antagonist) at various concentrations such that the final test serum- and sodium bicarbonate-free medium contained 100 μg/mL MWNTs. After chilling down to 4 °C, the cells were incubated for 1 h at 4 °C with test serum- and sodium bicarbonate-free medium that contained 100 μg/mL MWNTs, washed 3 times with serum- and sodium bicarbonate-free medium, and then washed 2 times with PBS. In all cases, the amounts of cell-surface bound MWNTs in the cell lysate samples were determined using the SDS-PAGE method, as described previously herein.

### 2.13. Immunofluorescence Microscopy of WT and SR-A1 Knockout RAW 264.7 Cells

A total of ~2 × 10^4^ RAW 264.7 cells were seeded on coverslips in 4-well plates and incubated in medium at 37 °C for 48 h to allow the cells to adhere to the plates. RAW 264.7 cells were incubated in serum-free medium for 1 h at 37 °C to deplete the serum in the cells. The cells were washed three times with media and 2 times with PBS. Then the cells were fixed with 4% paraformaldehyde at room temperature for 20 min followed by washing with PBS. The cells were incubated in blocking buffer containing 4% fish gelatin in PBS at room temperature for 1 hour to block non-specific protein-protein interactions. The cells were incubated with mouse SR-AI/MSR Alexa Fluor^®^ 488-conjugated antibody or a rat IgG2B Alexa Fluor^®^ 488-conjugated monoclonal antibody as the isotype control at room temperature for 1 h in the dark; control cells were not treated with any antibody. After rinsing, cell nuclei were stained with Hoechst 33342 dye for 10 min at room temperature. Then the cells were washed two times with PBS to remove excess dye. The coverslips were mounted on the glass slide using Fluoromount-G™. Images were taken with a Nikon Eclipse TE-2000 fluorescence microscope using a 60× oil-immersion objective with a NA of 1.4; the images for Hoechst 33342 (Ex. 350 nm; Em. 435–485 nm) and Alexa Fluor® 488 (Ex. 488 nm; Em. 520–550 nm) were overlaid using ImageJ software. 

### 2.14. Flow Cytometry for Surface Receptor(s) on WT and SR-A1 Knockout RAW 264.7 Cells

A total of ~2 × 10^6^ RAW 264.7 cells were seeded in 10-mm plates and incubated in medium at 37 °C for 48 h to allow the cells to adhere to the plates. The cells were rinsed and harvested with warm FACS staining buffer (1% BSA in PBS) in 15 mL centrifuge tube followed by centrifugation (1000× g) for 5 min. The cells were suspended in 1 mL of FACS staining buffer, then three 100 µL-aliquots of the cell suspension were used to determine cell counts in each aliquot using a Beckman Coulter particle counter. A total of ~1 × 10^6^ cells in 100 μL FACS staining buffer were aliquoted into 2 mL tubes. The cells were incubated in blocking buffer containing 5 μg IgG for 15 min at 4 °C to block non-specific protein interactions. The cells were stained with 5 μg mouse SR-AI/MSR Alexa Fluor^®^ 488-conjugated antibody (R&D Systems cat. No. FAB1797G) or a rat IgG2B Alexa Fluor^®^ 488-conjugated monoclonal antibody (R&D Systems cat. No. IC013G) as the isotype control for 30 min at 4 °C in the dark. Unbound antibody was removed by washing and re-suspending the cells in 1.5 mL FACS staining buffer thrice. The cells were re-suspended in 500 μL of FACS staining buffer for the final flow cytometric analysis. Flow cytometry analysis and data processing were performed using BD Accuri™ C6 Plus flow cytometer and CSampler™ Plus software (Becton and Dickinson Company, Franklin Lakes, NJ, USA) to determine the mean fluorescent index of each sample using a 518–548 nm emission filter.

## 3. Results

### 3.1. Characterization of BSA-MWNT Suspensions

The sonication and centrifugation protocol used to prepare purified BSA-coated MWNT suspensions is shown in Scheme 1. The initial baking step is to inactivate lipopolysaccharide derived from bacteria, should any be present. DLS and zeta potential analyses were used as part of a quality control routine for the preparation of all MWNT suspensions, as previously described [25,27]. Table 1 shows few differences in the particle size distributions of BSA-pMWNT and BSA-cMWNT suspensions, and that the zeta potentials for the BSA-cMWNTs in deionized water were slightly more negative than those for the BSA-pMWNTs. Zeta potentials were also determined for BSA-pMWNTs and BSA-cMWNTs in cell culture medium with and without 10% serum. In both matrices, the values were less negative for both MWNT samples in medium than in water as expected due to the increase in salt and/or serum proteins; the BSA-cMWNTs still had a slightly more negative zeta potential than the BSA-pMWNTs as expected due to the presence of ionized carboxyl groups on the cMWNTs.

### 3.2. BSA-pMWNTs and BSA-cMWNTs Are Not Significantly Toxic to RAW 264.7 Cells

**Figure 1:**
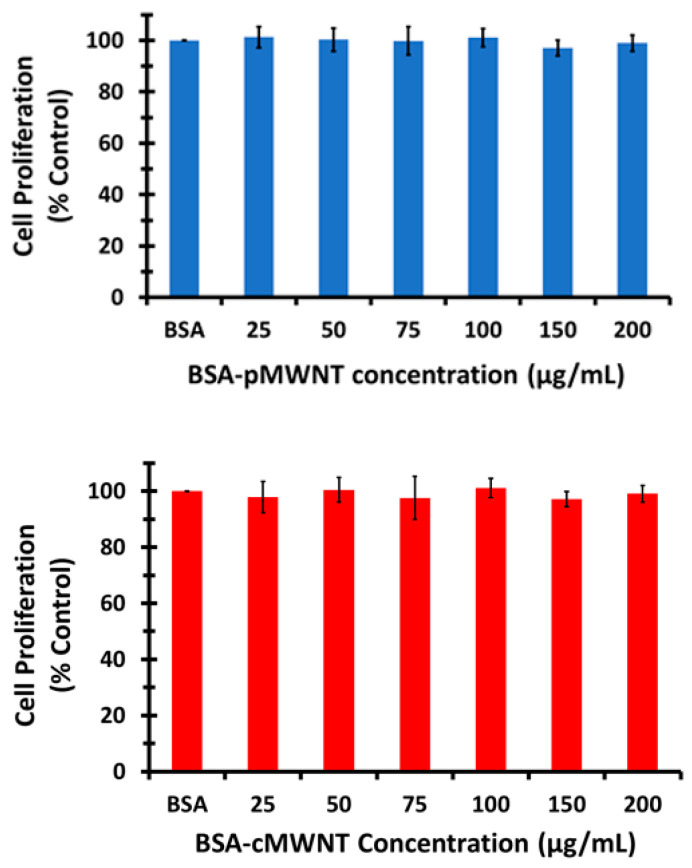
Cell proliferation of RAW 264.7 cells cultured with BSA-MWNTs. MWNTs suspended in a 0.10 mg/mL BSA working solution were mixed with an equal volume of 2X-concentrated medium to produce MWNT concentrations shown on the x-axes of the graphs. An equivalent number of cells were seeded in 48-well plates and incubated at 37 °C under standard cell culture conditions for 24 h prior to the experiment. Cell proliferation after incubation with control and test media for 24 h at 37 °C was determined by the crystal violet assay as described in the Methods, where the proliferation of control cells exposed to BSA in the absence of MWNTs was set to 100%. (**Top**) RAW 264.7 macrophage cell proliferation post 24-h incubation with various concentrations of BSA-pMWNTs. (**Bottom**) RAW 264.7 macrophage cell proliferation post 24-h incubation with various concentrations of BSA-cMWNTs. Both data sets are the mean of quadruple samples in three independent experiments ± the standard deviation (SD).

The cell proliferation of RAW 264.7 cells incubated with BSA-pMWNTs or cMWNTs was measured after 24-h exposure to different concentrations of MWNTs up to 200 µg/mL using a previously standardized crystal violet assay [28]. The control in each case was cells exposed to BSA alone. Figure 1 shows no significant decline in cell proliferation for RAW 264.7 cells with either BSA-pMWNTs or cMWNTs at the highest concentrations tested (200 µg/mL); however, exposures longer than 24 h could reveal toxicity. Except where noted, a MWNT concentration of 100 µg/mL was chosen for the majority of experiments involving a constant MWNT concentration.

### 3.3. Evidence for BSA-MWNT Receptors on RAW 264.7 Cells

**Figure 2:**
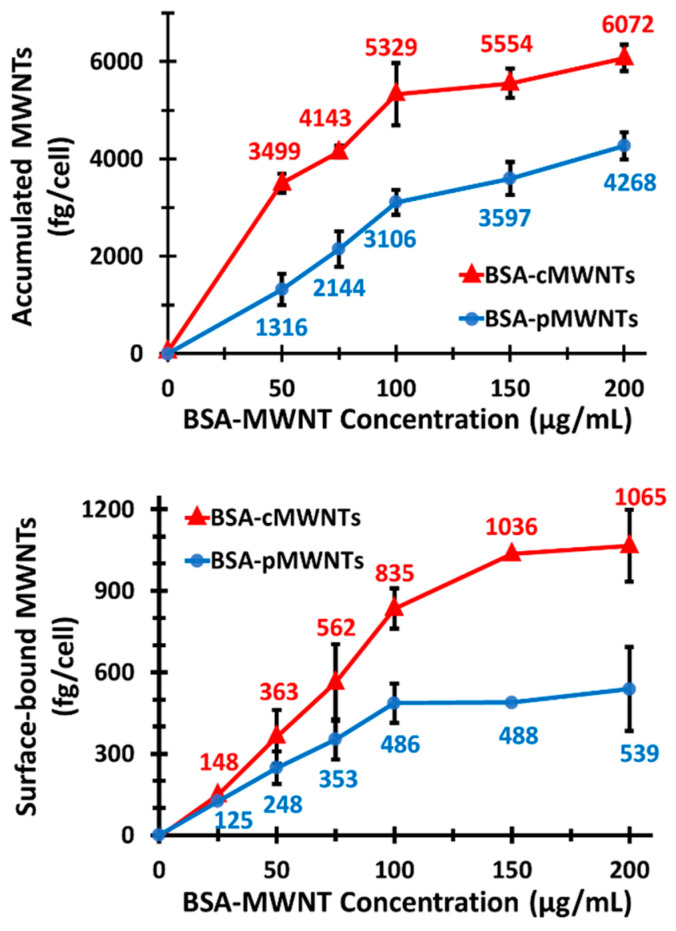
Accumulation at 37 °C and surface binding at 4 °C of BSA-MWNTs by RAW 264.7 cells as a function of the applied BSA-MWNT concentration. MWNTs suspended in a 0.10 mg/mL BSA working solution were mixed with an equal volume of 2X-concentrated medium to produce MWNT concentrations shown in the x-axes of the graphs. Exposure to a 0.10 mg/mL BSA working solution alone (in the absence of MWNTs) was the control. (**Top**) RAW 264.7 cells in 6-well plates were incubated at 37 °C for 24 h in complete medium with serum that contained either BSA alone, BSA-pMWNTs (blue line), or BSA-cMWNTs (red line). After incubation, MWNTs were extracted from cells and quantified by the SDS-PAGE method. (**Bottom**) Cells in 6-well plates were pre-incubated with serum-free medium (in the absence of BSA-MWNTs) for 2 h at 37 °C to deplete the serum in the cells. The cells were then pre-chilled to 4 °C and incubated at 4 °C for 1 h in serum- and sodium bicarbonate-free medium that contained either a 0.10 mg/mL BSA working solution without MWNTs, with BSA-pMWNTs (blue line), or with BSA-cMWNTs (red line). After incubation, surface-bound MWNTs were extracted and quantified by the SDS–PAGE method. For both data sets the numbers above the data points are the mean femtograms of MWNTs/cell; each data point is the mean of ≥3 independent experiments ± SD.

The accumulation of MWNTs by RAW 264.7 cells at 37 °C as a function of the applied BSA-MWNT concentrations between 0 and 200 μg/mL at 37 °C for 24 h was determined for BSA-pMWNTs and cMWNTs (Figure 2 top). For both, the uptake was linear to ~100 μg/mL and then began to decline as the concentration approached 200 μg/mL, consistent with a saturable receptor-mediated uptake process. To determine whether the receptors could be saturated when bound MWNTs were not internalized and in the absence of serum that otherwise could complicate the interpretation of the results, MWNT binding to cells was performed at 4 °C in medium without serum. RAW 264.7 cells were incubated with different concentrations of BSA-MWNTs (0–200 μg/mL) at 4 °C for 1 h in serum- and sodium bicarbonate-free medium. As shown in Figure 2 bottom, these experiments directly demonstrated that the binding of both MWNT types to the cell surface was a saturable function of the applied MWNT concentration, supporting the idea that there are receptors that bind BSA-coated MWNTs. Note also that more BSA-cMWNTs were bound than BSA-pMWNTs, suggesting that there are differences in the receptor interactions between the two MWNT types.

**Figure 3:**
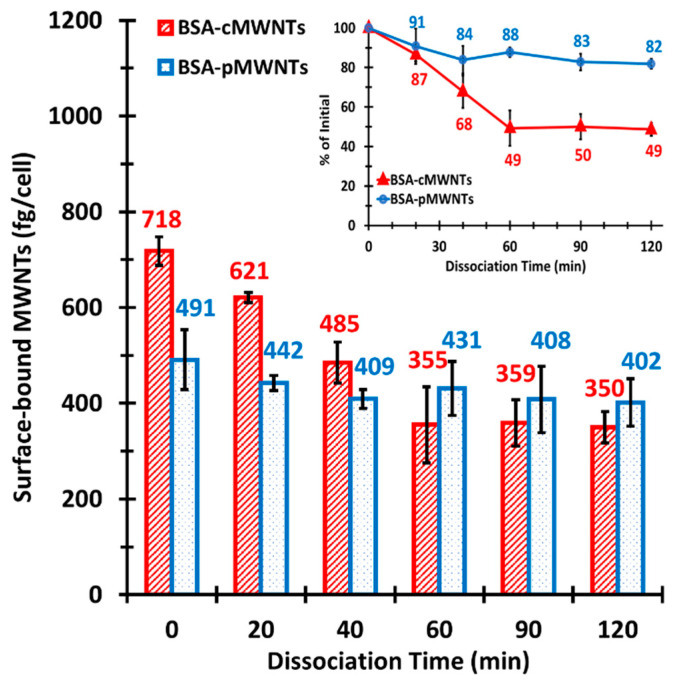
Dissociation of bound BSA-cMWNTs and BSA-pMWNTs from RAW 264.7 cells at 4 °C. A total of 100 µg/mL of BSA-cMWNTs or BSA-pMWNTs in serum- and sodium bicarbonate-free medium were incubated with RAW 264.7 cells at 4 °C for 1 h to achieve binding, then washed and incubated in serum- and sodium bicarbonate-free medium for the indicated times, as described in Methods. Surface-bound MWNTs were extracted and quantified by the SDS-PAGE method. The numbers above the bars are the mean femtograms of MWNTs/cell. **Inset:** The data are plotted as the percentage of the initial surface-bound MWNTs at t = 0 min. Data are the mean of ≥3 independent experiments ± SD.

To further characterize the ligand/receptor properties of bound BSA-coated MWNTs, the dissociation of bound BSA-cMWNTs and BSA-pMWNTs from cells was measured in the absence of serum at 4 °C. Briefly, RAW 264.7 cells were incubated with BSA-coated MWNTs to allow binding at 4 °C, washed, and further incubated in medium without serum to allow dissociation, followed by quantitating the amount of cell-bound MWNTs as a function of dissociation time. BSA-pMWNTs dissociated very slowly from cells, with more than 80% of the material still bound after 120 min (Figure 3, inset). This slow dissociation is not surprising considering that BSA is likely a major determinant of receptor interaction, and there are multiple copies of BSA on each nanotube that may simultaneously interact with multiple receptors, decreasing the probability of dissociation. BSA-cMWNTs’ dissociation was biphasic, with about 50% of the bound material dissociating within the first hour, followed by a slowly dissociating component, suggesting that BSA-cMWNTs may contain two binding sites for cells that have different dissociation rates from the two receptor sites. Further, the slowly dissociating component seen with BSA-cMWNTs might share features with the slowly dissociating material observed with BSA-pMWNTs. Regardless of mechanistic details, these data emphasize that the receptor interaction characteristics of BSA-cMWNTs and BSA-pMWNTs are not identical.

**Figure 4:**
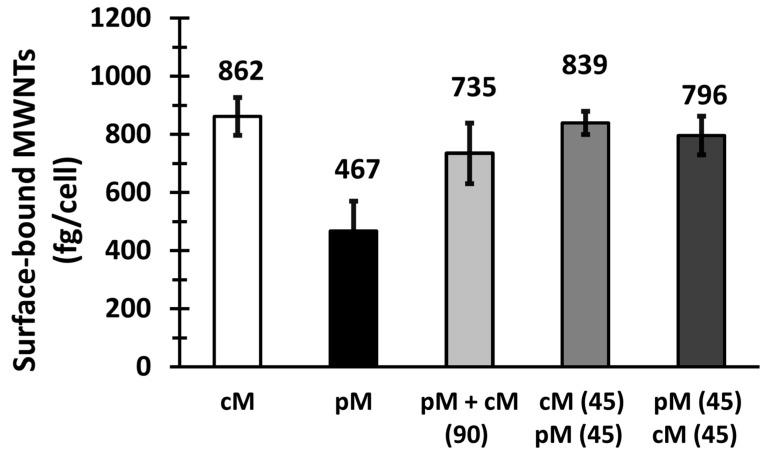
Test for additive binding of BSA-cMWNTs and BSA-pMWNTs to cells. Equivalent numbers of RAW 264.7 cells were seeded in 6-well plates and incubated at 37 °C under standard cell culture conditions for 24 h prior to the experiment in preparation for the additive binding studies as described in Methods. Cells were exposed to serum- and sodium bicarbonate-free media containing either 100 µg/mL BSA-pMWNTs or BSA-cMWNTs (labelled pM and cM in the graph) followed by incubation at 4 °C for 90 min to establish the amount of each bound when separate. Additive binding was tested by exposing the cells simultaneously to both BSA-cMWNTs and BSA-pMWNTs for 90 min. In a slightly different experimental design, the cells incubated with either BSA-cMWNTs or BSA-pMWNTs at 4 °C for 45 min, washed, and incubated with BSA-pMWNTs or BSA-cMWNTs, respectively, at 4 °C for 45 min for a total incubation time of 90 minutes. Surface-bound MWNTs were extracted and quantified using the SDS-PAGE method. The numbers above the bars are the mean femtograms of MWNTs/cell, and each data point is the mean of ≥3 independent experiments ± SD.

One explanation for the apparent differences between BSA-cMWNTs and BSA-pMWNTs in the number of cell surface binding sites and the differing dissociation kinetics is that there are two independent receptors on these cells—one for BSA-coated cMWNTs and another for BSA-coated pMWNTs. If so, then their binding should be additive at saturation; that is, if BSA-cMWNTs and BSA-pMWNTs are both added simultaneously, the total cell-associated MWNTs should be the sum of the amount for each when added alone. As shown in Figure 4, when cells were incubated with both BSA-cMWNTs and BSA-pMWNTs, the amount bound by cells was greater than for BSA-pMWNTs alone, but did not exceed that of BSA-cMWNTs alone, which is not fully additive. In a slightly different experimental design to test additive binding, the cells were exposed to the ligands sequentially—an experimental design that avoids the possible interaction of cMWNTs and pMWNTs when they are together in medium during binding. Cells were first exposed for 45 min to BSA-cMWNTs alone, followed by washing and exposure for 45 min to BSA-pMWNTs. The order of the two sequential ligand additions was then reversed, with results seen in the last two bars of Figure 4. When BSA-cMWNTs were added first, followed by BSA-pMWNTs, there was no additional binding compared to BSA-cMWNTs alone, suggesting that there were no further open sites for BSA-pMWNTs. When BSA-pMWNTs were added first, followed by BSA-cMWNTs, there was additional binding compared to pMWNTs alone, but binding did not exceed that of BSA-cMWNTs alone. Altogether, these data do not fit a simple model of additive binding with two independent receptors each interacting autonomously with the two ligands. Rather, they suggest a semi-additive situation where BSA-cMWNTs can occupy all the sites that BSA-pMWNTs may interact with, but that there are sites for BSA-cMWNTs to which BSA-pMWNTs do not bind.

### 3.4. An SR-A Antagonist Reduces Binding of BSA-MWNTs to RAW 264.7 Cells

**Figure 5:**
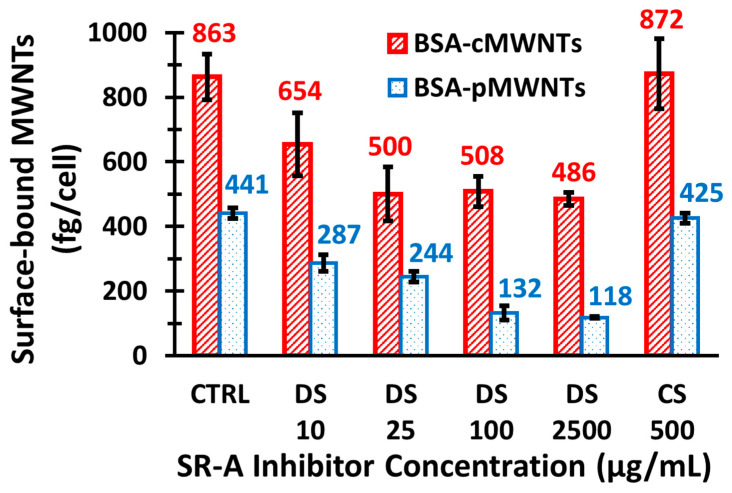
Effect of dextran sulfate on BSA-MWNT binding to RAW 264.7 cells at 4 °C. MWNTs suspended in a 0.10 mg/mL BSA working solution were mixed with an equal volume of 2X-concentrated, serum- and sodium bicarbonate-free medium to give a MWNT concentration of 100 µg/mL followed by the addition of chondroitin sulfate (CS) or the SR-A1 antagonist dextran sulfate (DS) at various concentrations as described in the Methods. The serum- and sodium bicarbonate-free medium control contained the same 100 µg/mL BSA-MWNTs, but without CS or DS. After initial plating and attachment to the substrate, cells were pre-incubated with serum-free medium (in the absence of MWNTs) for 2 h at 37 °C to deplete the serum in the cells. The cells were then pre-chilled to 4 °C and incubated at 4 °C for 1 h in serum- and sodium bicarbonate-free test medium that contained either a 0.10 mg/mL BSA working solution without MWNTs, with BSA-pMWNTs ± DS or CS (blue bars and line), or with BSA-cMWNTs ± DS or CS (red bars and line). Surface-bound MWNTs were extracted and quantified using the SDS-PAGE method. The numbers above the bars are the mean femtograms of MWNTs/cell. Data are the mean of ≥3 independent experiments ± SD.

SR-As are involved in the binding of anionic ligands and certain modified proteins, such as oxidized LDL and maleylated albumin [30,31,32,33]. Moreover, the interaction of BSA with several nanoparticles causes conformation changes in BSA that expose cryptic SR-A1 binding sites [34,35,36]. In addition, there is indirect evidence that SRs bind carbon nanotubes [20]. Work from our lab also provided evidence that PF108-cMWNTs, but not PF108-pMWNTs, interact with SR-A1 [12,21]. Thus, SR-A1 is a potential receptor for BSA-MWNTs. This was initially explored by determining whether dextran sulfate, a known antagonist of SR-As, interferes with the binding of BSA-coated MWNTs. Chondroitin sulfate, an anionic polysaccharide that is not a SR-A1 inhibitor, was used as the control. RAW 264.7 cells were exposed to 100 μg/mL of BSA-MWNTs in serum- and sodium bicarbonate-free medium at 4 °C in the presence or absence of dextran sulfate or chondroitin sulfate, as indicated in Figure 5. The amount of BSA-cMWNTs bound to the cells declined as a function of dextran sulfate concentration and leveled off to about 50% compared to cells not exposed to the antagonist, whereas the amount of BSA-pMWNTs bound appeared to monotonically decline to a final level of ~25% of the control at the highest dextran sulfate concentration. These data again emphasize the differences in the receptor binding properties of the two BSA-MWNT types and further suggest that binding of both MWNTs types to receptors are sensitive to an SR-A1 antagonist; however, interpreting the data is not straightforward because the inhibition was partial, especially for BSA-cMWNTs. Therefore, studies were performed with cells that over- or under-express SR-A1 to clarify whether SR-A1 might interact with BSA-cMWNTs or BSA-pMWNTs, or both.

### 3.5. Evidence That SR-A1 Mediates the Uptake of Both BSA-cMWNTs and BSA-pMWNTs in CHO Cells Overexpressing SR-A1

**Figure 6:**
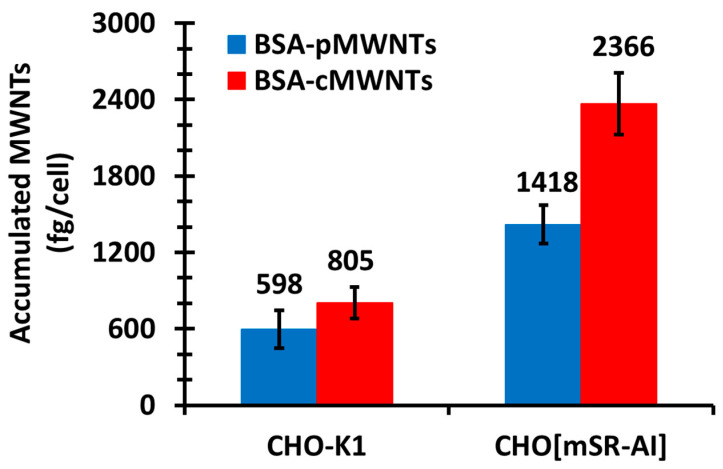
Accumulation of BSA-MWNTs by wild-type Chinese hamster ovary (WT CHO)-K1 control cells and Chinese hamster ovary (CHO) cells that overexpress SR-A1 receptors (CHO[mSR-AI] cells) at 37 °C. Equivalent numbers of each cell line were seeded in 6-well plates and incubated at 37 °C under standard cell culture conditions for 24 h prior to the experiment. The cells were then incubated at 37 °C for 24 h in medium that contained BSA-pMWNTs (blue bars) or BSA-cMWNTs (red bars) each at 100 µg/mL. After incubation, MWNTs were extracted from the cells and quantified by the SDS-PAGE method. The numbers above the data points are the mean femtograms of MWNTs/cell, and each data point is the mean of ≥3 experiments ± SD.

CHO cells stably transfected with mouse SR-A1 cDNA (CHO[mSR-AI] cells) [24] were studied to determine whether the expression of SR-A1 in a cell line that does not normally express the receptor results in the accumulation of BSA-coated MWNTs by the cells. CHO[mSR-AI] cells overexpressing SR-A1 were incubated at 37 °C for 24 h with 100 μg/mL of BSA-pMWNTs or cMWNT dispersions. Similarly treated wild-type CHO-K1 cells were the control. The results showed that the SR-A1 overexpressing CHO-K1 cells accumulated BSA-pMWNTs and BSA-cMWNTs two and three times more, respectively, compared to the control cells (Figure 6). This evidence supports the idea that SR-A1 is a receptor for both BSA-cMWNTs and BSA-pMWNTs, and also recapitulates the observation in Figure 2 that BSA-cMWNTs were accumulated to a greater extent than BSA-pMWNTs.

### 3.6. SR-A1 Knockout RAW 264.7 Cells Bind and Accumulate Far Less BSA-MWNTs Than WT Cells

**Figure 7:**
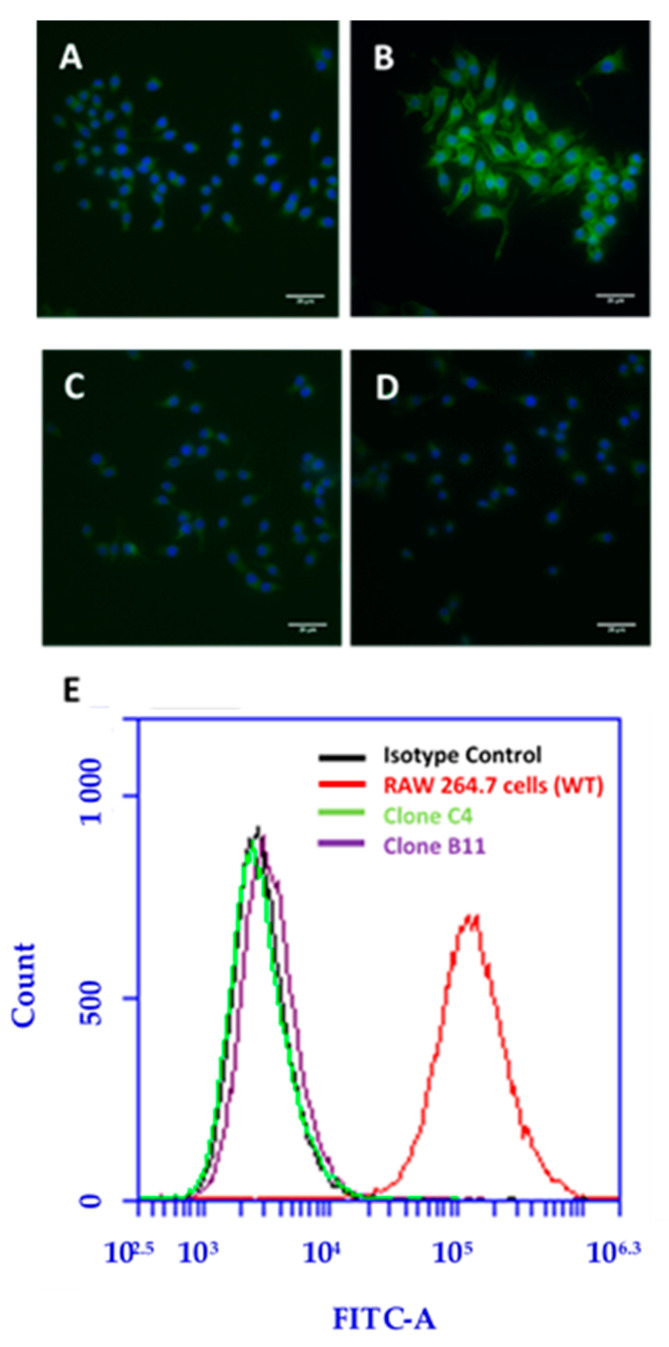
Immunofluorescence microscopy and flow cytometric analysis of WT and SR-A1 knockout RAW 264.7 cells. Immunofluorescence images of **(A)** control WT RAW 264.7 cells without mouse anti-SR-AI/MSR Alexa Fluor^®^ 488-conjugated antibody, **(B)** WT RAW 264.7 cells incubated with mouse anti SR-AI/MSR Alexa Fluor^®^ 488-conjugated antibody, and (**C**,**D**) two different clones (C4 and B11) of SR-A1 knockout RAW 264.7 cells incubated with anti-mouse SR-AI/MSR Alexa Fluor^®^ 488-conjugated antibody. Hoechst 33342 staining is shown in blue and Alexa Fluor^®^ 488 staining is shown in green. All images are normalized to the same intensity scale, and the scale bars represent 20 μm. **(E)** Flow cytometry analyses where the black line represents WT RAW 264.7 cells incubated with the rat IgG2B Alexa Fluor^®^ 488-conjugated monoclonal antibody as the isotype control, the red line represents WT RAW 264.7 cells incubated with mouse anti-SR-AI/MSR Alexa Fluor^®^ 488-conjugated antibody, and the green and purple lines represent two different clones (C4 and B11, respectively) of SR-A1 knockout RAW 264.7 cells incubated with mouse anti-SR-AI/MSR Alexa Fluor^®^ 488-conjugated antibody. The x-axis denotes fluorescence detected in the 518–548 nm spectral region, and the y-axis denotes the number of events for each analysis.

Another approach to understanding the role that SR-A1 has in the uptake and binding of BSA-MWNTs is to knock out the SR-A1 gene using CRISPR-Cas9 technology. A RAW 264.7 cell knockout pool was obtained that contained a high proportion of cells with a mutation in the SR-A1 gene at a site near the beginning of the DNA sequence. This ensured that both SR-A1 as well as SR-A1.1 protein expression would be affected. A dilution cloning strategy was used to obtain 10 cell clones that did not express SR-A1 receptors on their surface as validated by immunofluorescence microscopy and flow cytometry. Both techniques showed that WT RAW 264.7 cells had high expression of SR-A1 receptors, whereas two knockout clones selected for study (termed C4 and B11) had negligible surface receptors (Figure 7).

**Figure 8:**
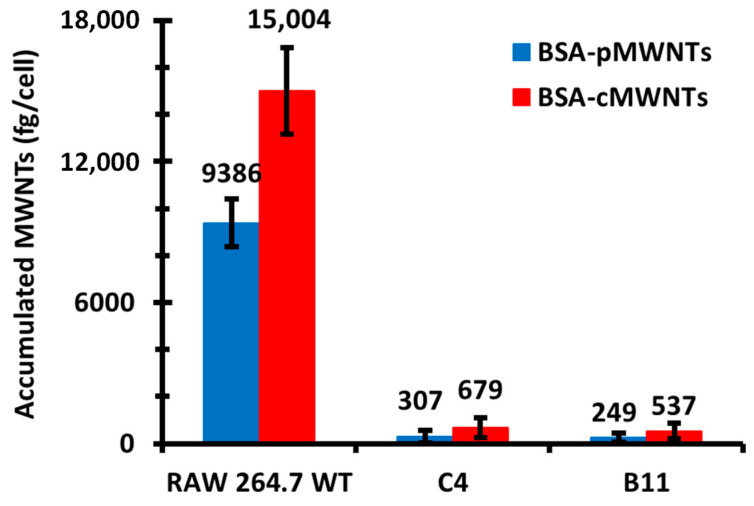
Accumulation of BSA-MWNTs by WT and SR-A1 knockout RAW 264.7 cells at 37 °C. MWNTs suspended in a 0.10 mg/mL BSA working solution were mixed with an equal volume of 2X-concentrated medium to produce MWNT concentrations of 100 µg/mL. Equivalent numbers of WT and SR-A1 knockout RAW 264.7 cells were seeded in 6-well plates and incubated at 37 °C under standard cell culture conditions for 24 h prior to the experiment. The cells were then incubated at 37 °C for 24 h in medium that contained BSA-pMWNTs (blue bars) or BSA-cMWNTs (red bars). After incubation, MWNTs were extracted from cells and quantified by the SDS-PAGE method. The numbers above the data points are the mean femtograms of MWNTs/cell. Data are the mean of ≥3 experiments ± SD.

To assess the recognition of BSA-pMWNTs and BSA-cMWNTs by SR-A1 receptors, the accumulation of 100 μg/mL BSA-coated pMWNTs or cMWNTs was measured using knockout clones C4 and B11 with the corresponding WT RAW 264.7 cells for comparison. The cells were incubated at 37 °C for 24 h with 100 μg/mL BSA-MWNTs and the accumulated MWNTs were measured using SDS-PAGE. As shown in Figure 8, the amount of accumulated MWNTs in the knockout SR-A1 cell lines was significantly decreased for both BSA-pMWNTs and BSA-cMWNTs compared to the WT RAW 264.7 cells.

**Figure 9:**
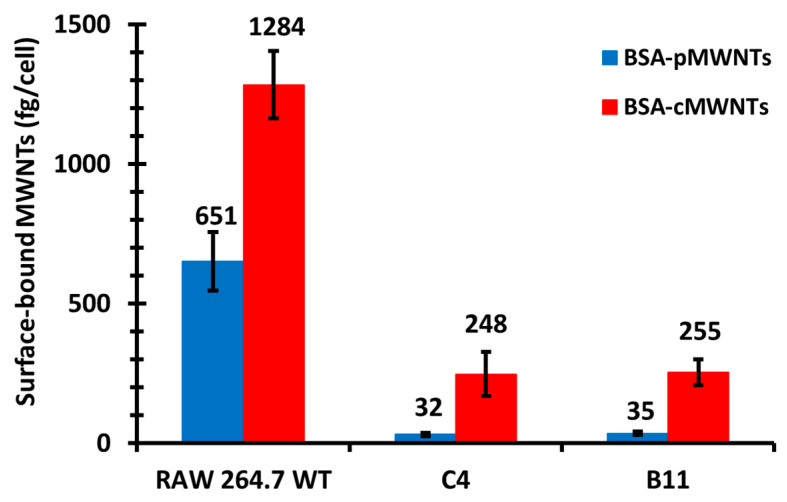
Surface binding of BSA-MWNTs by WT and SR-A1 knockout RAW 264.7 cells at 4 °C. MWNTs suspended in a 0.10 mg/mL BSA working solution were mixed with an equal volume of 2X-concentrated serum- and sodium bicarbonate-free medium to produce MWNT concentrations of 100 µg/mL. Equivalent numbers of WT and SR-A1 knockout RAW 264.7 cells were seeded in 6-well plates and incubated at 37 °C under standard cell culture conditions for 24 h prior to the experiment. Next, the cells were pre-incubated with serum-free medium (in the absence of BSA-MWNTs) for 2 h at 37 °C to deplete the serum in the cells. The cells were then pre-chilled to 4 °C and incubated at 4 °C for 1 h in serum- and sodium bicarbonate-free medium that contained either BSA-pMWNTs (blue bars) or BSA-cMWNTs (red bars). After incubation, surface-bound MWNTs were extracted and quantified by the SDS-PAGE method. Numbers above the data points are the mean femtograms of MWNTs/cell. Data are the mean of ≥3 independent experiments ± SD.

The binding of 100 μg/mL BSA-coated cMWNTs and pMWNTs by RAW 264.7 cells was also studied using the same knockout SR-A1 clones (C4 and B11) and corresponding WT RAW 264.7 cells at 4 °C in the absence of serum, conditions under which MWNT binding by macrophages can be directly measured where the influence of protein coronas and cell uptake are controlled. The results indicated that there is a significant decrease in binding of BSA-pMWNTs and BSA-cMWNTs by SR-A1 knockout RAW 264.7 cells compared to WT RAW 264.7 cells (Figure 9). Interestingly, 20% of the surface-bound BSA-cMWNTs were still present on the SR-A1 knockout cells, suggesting that a low binding capacity for BSA-cMWNTs still remained. Taken together, the observation that CHO cells expressing SR-A1 do bind BSA-MWNTs and the finding that RAW 264.7 cells lacking SR-A1 have greatly reduced binding, suggest that SR-A1 has a dominant role in the binding and accumulation of both BSA-MWNTs types.

## 4. Discussion

WT RAW 264.7 cells accumulated both BSA-cMWNTs and BSA-pMWNTs as a function of concentration after a 24 h exposure at 37 °C, although BSA-coated cMWNTs were accumulated to almost twice the amount of pMWNTs at each concentration tested. Uptake for both was near linear up to 100 µg/mL, after which the rate of accumulation was reduced. The break in the uptake curve at 100 µg/mL suggests a saturable receptor could be involved in the uptake process; however, accumulation depends not only on uptake, but also on potential loss of the MWNTs from cells by either recycling or degradation, or a loss of surface receptors that are internalized from the cell surface but not replaced. To focus on the initial interaction of MWNTs with cells, binding experiments were performed at 4 °C where internalization is inhibited. Moreover, serum proteins other than BSA that might confound the interpretation of the results were absent from the binding medium. Under these conditions, the binding of BSA-coated cMWNTs or pMWNTs to RAW 264.7 cells was near linear up to 100 µg/mL and then began to plateau, suggesting a saturable receptor-mediated binding event. There were two notable observations in comparing the binding of BSA-coated MWNTs to that we previously described for PF108-coated MWNTs. First, BSA-pMWNTs bound to cells, whereas previous studies showed that PF108-coated pMWNTs did not [12,21]. This indicates that the BSA corona confers the ability of pMWNTs to bind cells. Second, the cells bound more BSA-cMWNTs than BSA-pMWNTs, evidence that there remains a difference in binding capacity between the two MWNT types. Differences between BSA-cMWNTs and BSA-pMWNTs were also seen in their kinetics of dissociation from cells: BSA-pMWNTs dissociated very slowly, whereas BSA-cMWNTs had a faster dissociating component followed by a slowly dissociating component.

One model to explain the difference in the binding of BSA-cMWNTs and BSA-pMWNTs to cells is that there are two independent receptors—one for each type of MWNT. If there are two receptors interacting independently with two ligands, then exposing cells simultaneously to both ligands should result in an amount bound that is the sum of both when added separately. However, this was not observed. The amount bound after simultaneous exposure to both BSA-cMWNTs and BSA-pMWNTs never exceed the amount bound to cells when BSA-cMWNTs were added alone, which is not a simple additive result. To further explore this issue, sequential binding experiments were undertaken. The level of cell-associated MWNTs when BSA-cMWNTs were added first, followed by BSA-pMWNTs, was equal to the amount of MWNTs bound when BSA-cMWNTs were added alone, which is not additive. However, when the order was reversed and BSA-pMWNTs were added first followed by BSA-cMWNTs, there was more binding than observed when BSA-pMWNTs were added alone, and the amount was again equal to the increased binding seen with BSA-cMWNTs alone, an additive result. Altogether, the results of the binding experiments suggest a semi-additive model: BSA-cMWNTs can occupy all the binding sites available to BSA-pMWNTs, plus additional sites not available to BSA-pMWNTs. Thus, when BSA-cMWNTs are added first, no binding of BSA-pMWNTs occurs because the sites are occupied by BSA-cMWNTs. However, when BSA-pMWNTs are added first, there remain sites available for BSA-cMWNTs to which BSA-pMWNTs cannot bind. 

The semi-additive data are compatible with a two-receptor model and also with a model where a single receptor has two binding sites. In the two-receptor model, one receptor would bind both cMWNTs and pMWNTs, and the other receptor would bind only cMWNTs. To help address the question of whether one or two receptors were involved in binding cMWNTs and pMWNTs, the accumulation and binding of BSA-coated MWNTs was studied with RAW 264.7 cells in which the SR-A1 gene had been knocked out. Two clones isolated from the knockout pool, which were shown to lack immunologically detectable SR-A1 on their surfaces, failed to accumulate either BSA-coated cMWNTs or pMWNTs at 37 ℃. In binding studies at 4 ℃, the binding of BSA-pMWNTs was negligible and the binding of BSA-cMWNTs was reduced by 80%. It is not clear what is responsible for the 20% of BSA-cMWNT binding in the knockout cells, but perhaps one or more minor receptors for BSA-cMWNTs are present at low levels, and their contributions are seen in SR-A1 knockout cells. Nevertheless, it appears that knocking out SR-A1 severely affects the accumulation and binding of both BSA-cMWNTs and BSA-pMWNTs. 

The simplest explanation for the knockout results is that SR-A1 is a receptor for both BSA-cMWNTs and BSA-pMWNTs. However, an alternative explanation is that knocking out SR-A1 suppresses the expression of one or more other cell surface proteins that could be major receptors for BSA-coated MWNTs. Two lines of evidence argue against this possibility. One is that dextran sulfate, a known antagonist of ligand binding to SR-A1, at least partially inhibited the binding of both BSA-coated pMWNTs and cMWNTs to cells, supporting the idea that SR-A1 is a receptor for these ligands. Second, CHO-K1 cells that ectopically express SR-A1 accumulated significantly more BSA-coated cMWNTs and pMWNTs than normal CHO-K1 cells. It seems unlikely that a covert receptor is activated in CHO cells, a cell type very different than RAW 264.7 macrophages, upon expression of SR-A1. Altogether, the simplest interpretation of the evidence argues that SR-A1 binds both BSA-cMWNTs and BSA-pMWNTs. 

Understanding what features of BSA-coated MWNTs interact with SR-A1 is an interesting challenge. Previous work established that PF108-coated cMWNTs bound to and were accumulated by macrophages that expressed SR-A1 in the absence of serum or serum proteins [12], whereas alveolar macrophages derived from mice knocked out for SR-A1 failed to accumulate the MWNTs [21]. PF108-coated pMWNTs were not bound or accumulated by either SR-A1 positive or negative macrophages [12,21]. Thus, no protein corona was necessary for SR-A1 to interact with cMWNTs. This suggested that one or more oxidized functionalities intrinsic to cMWNTs (carboxyl, hydroxyl, phenolic, etc.) are structural features potentially recognized by SR-A1. SR-A1 access to cMWNT surface features might occur at nanotube ends where the high curvature may not support coat binding and where oxidized functionalities are often located due to ring strain [37,38,39,40]. In addition, the residence time of BSA on MWNTs appears to be short and not all the surface is covered with protein at one time [41]. Thus, it is likely that SR-A1 would have access to oxidized groups intrinsic to the MWNT surface of BSA-coated cMWNTs. 

**Figure 10:**
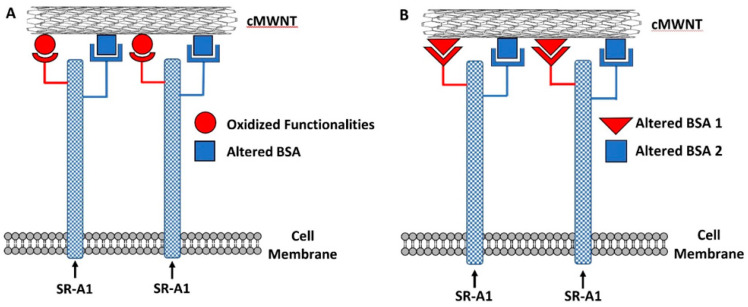
Models for the binding of cMWNTs to SR-A1. (**A**) Model where one site on SR-A1 interacts with altered binding sites on a BSA conformer and another site interacts with oxidized functionalities on cMWNTs. (**B**) Model where one site on SR-A1 interacts with altered binding sites on BSA Conformer 1 and another site interacts with altered binding sites on BSA Conformer 2.

It is understood now that while native BSA does not interact with SR-A1, conformational changes in BSA upon binding several types of nanoparticles uncover latent sites that do bind SR-A1 [34,35,36]. Moreover, BSA undergoes significant conformation changes upon binding to cMWNTs [42]. This leads to Binding Hypothesis 1 in Figure 10A, where BSA-coated cMWNTs present two sites that can interact with SR-A1—one for oxidized groups inherent to the nanotube and another for the coat of conformationally altered BSA protein. This model may explain why more BSA-cMWNTs bind cells than BSA-pMWNTs, and also is consistent with the semi-additive binding data: all binding sites are occupied by BSA-cMWNTs, whereas only the BSA binding sites are occupied by BSA-pMWNTs. The model is also consistent with the differences in dissociation of the two MWNT types from cells assuming BSA-cMWNTs and BSA-pMWNTs bound to SR-A1 at BSA binding sites dissociate slowly and that BSA-cMWNTs bound to oxidized functionalities dissociates more rapidly.

An alternative model is one in which all the oxidized binding sites on cMWNTs are unavailable because they are covered by BSA, and that binding of BSA to cMWNTs exposes additional latent SR-A1 binding sites that are not exposed when BSA binds to pMWNTs; hence, cells bind more BSA-cMWNTs than BSA-pMWNTs. A model of this type shown in Figure 10B cannot be ruled out with the available data. 

SR-A1 is a homotrimer and each monomer comprises an N-terminal cytoplasmic tail, a transmembrane domain, a spacer region, an α-helical coiled coil domain, a collagenous domain, and a C-terminal scavenger receptor cysteine rich (SRCR) domain [15,30,43]. Depending on the ligand, either the collagenous domain, the SRCR, or both, may be involved in ligand binding of various scavenger receptors, but the details are not well understood and appear to depend on the structural context within each receptor type. For example, there is evidence from mutational studies with SR-A1 that positively charged residues in the collagenous domain are important for binding oxidized LDL [31,44]. Further, SR-A1.1, an alternatively spliced variant of SR-A1 lacking the SRCR domain, still binds oxidized LDL, suggesting that the collagenous domain is the major binding site for this ligand, although this does not rule out that the SRCR domain of SR-A1 may also interact with oxidized LDL or other protein ligands. Indeed, recent work suggests that the SR-A1 SRCR domain binds spectrin [45] and ferritin [46]. The SRCR domain is involved in the ligand binding by MARCO, a member of the class A scavenger receptors that shares the highly conserved SRCR domain with SR-A1 [47,48,49]. The functional unit of many scavenger receptor family members is a trimer, including SR-A1, and the potential for three ligand binding sites per trimer is believed to enhance binding avidity to larger ligands, such as intact bacteria, and which would presumably include large ENPs such as MWNTs [33]. This feature is not explicitly shown in the models of Figure 10, but could be accommodated. Nevertheless, given the intricacies of how different domains in scavenger receptors interact with ligands, it is difficult to parse which SR-A1 domains interact with what features of BSA-coated MWNTs. 

Additional complexities in scavenger receptor interaction with ligands arise from evidence that scavenger receptors, including SR-A1, may form complexes with other pattern recognition receptors, termed co-receptors, that also interact with the same ligand. The resulting complexes can then recruit components to form “Signalosomes” that contain two or more receptors bound to the same ligand plus associated signaling components that may activate cell signaling pathways [32,33,50]. For example, there is evidence from computational work [51] and from molecular docking studies that SWNTs may bind toll-like receptor 4 (TLR4) [52]. It would be interesting to know whether the ~20% of cMWNT binding to RAW 264.7 cells lacking SR-A1 seen in Figure 9 is due to TLR4. Thus, the simple models in Figure 10 may not capture the range of possibilities for how MWNTs interact with SR-A1 and other cell components via co-receptors. Nevertheless, SR-A1 is a key player evidenced by the major loss of binding in SR-A1 knockout cells and the gain of binding in CHO cells that ectopically express SR-A1.

## 5. Conclusions

From previous work, PF108-coated pMWNTs fail to bind to macrophages but BSA-coated pMWNTs do bind, suggesting that a BSA corona confers the ability of pMWNTs to bind to cells. Therefore, in this article we studied the interaction of BSA-MWNTs with macrophages using a direct binding assay under highly controlled conditions where the influence of nanotube functionalization and protein coronas could be controlled. The results demonstrated that the binding of both BSA-cMWNTs and BSA-pMWNTs to the cell surface was a dose-dependent and saturable function of the applied MWNT concentration. Both MWNT types bound and were accumulated by RAW 264.7 cells; however, the cells bound and accumulated two times more BSA-cMWNTs than BSA-pMWNTs, suggesting that there are more binding sites on the cell surface for BSA-cMWNTs than BSA-pMWNTs. The binding of BSA-coated cMWNTs and pMWNTs to RAW 264.7 cells was semi-additive, suggesting that a single receptor with two distinct binding sites could explain the data. SR-A1 knockout RAW 264.7 cells had significantly reduced binding and accumulation of both BSA-pMWNTs and cMWNTs and CHO cells that ectopically expressed SR-A1 accumulated both MWNT types, whereas WT CHO cells did not, suggesting that SR-A1 is the key receptor for both MWNT types. Models consistent with the data are proposed where SR-A1 has two binding sites that interact with BSA-coated MWNTs differently depending on the presence of a BSA corona and on the presence or absence of oxidized groups on the MWNTs. The approaches and observations in this study may contribute to the rational design of nanotoxicity remediation efforts and biomedical applications of engineered carbon nanoparticles.

## Figures and Tables

**Table 1:** Dynamic light scattering (DLS) particle size and zeta potential analyses of BSA-MWNT suspensions.

MWNT Suspension	DLS ^1^		Zeta Potential ^2^ (mV)		
	HDD (nm)	PDI	Water	Medium + FBS	Medium − FBS
**BSA-pMWNTs**	83.23	0.20	−31.2	−5.7	−5.6
**BSA-cMWNTs**	84.18	0.19	−32.5	−6.6	−6.1

^1^ Aliquots of purified pristine multi-walled carbon nanotubes (pMWNT) or carboxylated multi-walled carbon nanotubes (cMWNT) suspensions were diluted 1:10 in 0.10 mg/mL BSA working solutions. HDD is the hydrodynamic diameter, and PDI is the polydispersity index. ^2^ Aliquots of purified pMWNT or cMWNT suspensions were diluted 1:10 in deionized water, medium with fetal bovine serum (FBS), or FBS-free medium.

## Data Availability

The data presented in this study are available in Huynh, M.T.; Mikoryak, C.; Pantano, P.; Draper, R. Scavenger Receptor A1 Mediates the Uptake of Carboxylated and Pristine Multi-Walled Carbon Nanotubes Coated with Bovine Serum Albumin. *Nanomaterials* 2021, 11, 539, doi:10.3390/nano11020539.

## Authors’ Note

A companion paper has also been published in the Nanomaterials Special Issue “Nanoparticle-Macrophage Interactions: Implications for Nanosafety and Nanomedicine”: Wang, R.; Lohray, R.; Chow, E.; Gangupantula, P.; Smith, L.; Draper, R. Selective Uptake of Carboxylated Multi-Walled Carbon Nanotubes by Class A Type 1 Scavenger Receptors and Impaired Phagocytosis in Alveolar Macrophages. *Nanomaterials (Basel)* **2020**, *10*, 2417. doi:10.3390/nano10122417.
